# Testing variations between starters and substitute players in terms of total distance, high-speed running, and sprinting distance: a descriptive study on professional male soccer players

**DOI:** 10.5114/biolsport.2024.131817

**Published:** 2023-06-10

**Authors:** Janusiak Marcin, Ana Filipa Silva, Rui Silva, Aleksander Kosendiak, Bartłomiej Bogdański, Małgorzata Smoter, Gibson Praça, Filipe Manuel Clemente

**Affiliations:** 1 University WSB Merito, Wrocław, Poland; 2 Escola Superior Desporto e Lazer, Instituto Politécnico de Viana do Castelo, Rua Escola Industrial e Comercial de Nun’Álvares, 4900-347 Viana do Castelo, Portugal; 3 Research Center in Sports Performance, Recreation, Innovation and Technology (SPRINT), 4960-320 Melgaço, Portugal; 4 The Research Centre in Sports Sciences, Health Sciences and Human Development (CIDESD), 5001-801 Vila Real, Portugal; 5 Medicus Clinic, Physiotherapy Department, Wrocław, Poland; 6 Doctoral School, Gdansk University of Physical Education and Sport, Gdańsk, Poland; 7 Department of Basics of Physiotherapy, Gdansk University of Physical Education and Sport, Gdańsk, Poland; 8 School of Physical Education, Physiotherapy and Occupational Therapy, Federal University of Minas Gerais, Av. Antônio Carlos, 6627, CEP 31270-901, Belo Horizonte 31270-901, Brazil; 9 Instituto de Telecomunicações, Delegação da Covilhã, Lisboa 1049-001, Portugal

**Keywords:** Football, Match analysis, Load monitoring, Athletic performance

## Abstract

The purpose of this study was three-fold: (i) to compare total distance, high-speed running (HSR) distance, and sprint distance covered per 5-minute epoch by players acting as both starters and substitutes; (ii) to compare the locomotor demands between the moments the players entered the match (45–60, 60–75 and 75–90 minutes); and (iii) to compare the locomotor demands of the players between the variations of the within- and between-playing positions. Twenty-one male professional soccer players competing in the Professional Premier League of one of the European countries were observed over sixteen official matches. The players were monitored during all matches using a Global Navigation Satellite System. The measures collected were total distance (TD; m), distance in HSR, sprint distance, HSR, and sprint counts. Considering the comparisons between the splits over the second half of match play, a significant difference between the starters and the substitutes was observed only for sprint distance in the 90–95 minute split (Z = –2.023; p = 0.043). Moreover, no substantial differences were found between the moment the substitute player entered the match regarding total distance (H = 2.650; p = 0.266), HSR distance (H = 1.738; p = 0.419), and sprint distance (H = 0.048; p = 0.976). However, the comparison of between-playing positions revealed considerable differences in total distance (H = 29.246; p < 0.001), and HSR distance (H = 12.153; p = 0.002) covered by the players acting as starters. In contrast, for substitute players, such differences were reported in HSR distance (H = 27.892; p < 0.001) and sprint distance (H = 15.879; p < 0.001). In conclusion, this study suggests that acting as a starter or a substitute does not significantly affect the intensity of effort except during the last periods of match play. However, the contextual factor of performing in a specific playing position plays a significant role both for starters and substitutes.

## INTRODUCTION

The match running demands of professional men’s soccer assume a preponderant role in the training process, requiring consistent and objective monitoring on a week-by-week basis [1]. Soccer players are exposed to high loads during a match, and there can be a discrepancy betweenstarters and non-starters [2]. Therefore, appropriate match load monitoring can help coaches better adjust training programmes to players and to improve recovery mechanisms for their players [3].

Load monitoring is commonly categorized into two different and interconnected dimensions [4]: (i) external load (physical demands imposed on players); and (ii) internal load (psychobiological response to a given external load imposed). The load can be quantified through the use of objective measures, such as heart rate monitors, and/or through subjective measures, including the rate of perceived exertion (RPE). The external load, however, can be assessed using only objective measures, namely Global Navigation Satellite System (GNSS), accelerometers, and inertial measurement units (IMUs) [5]. Modern GNSS have accelerometers, and IMUs are integrated into one device. Given that, coaches can monitor a variety of locomotor measures during both training and matches, which allows them to adjust their training programmes to theirplayers’ needs [6].

The most common measures of external load quantified by GNSS include [6]: (i) distances covered at different speed thresholds; (ii) actions associated with changes in speed, such as accelerations and decelerations and/or changes in direction; and (iii) actions measured by the IMUs, such as impacts [6]. The distances covered at different speed thresholds can present significant variations depending on tactical behaviors during a match, a match half, and a field position of each player [7]. On the other hand, accelerometry-based measures can be more dependent on the dynamics of a match and are associated with higher injury incidence [8].

In the last few years there has been increasing evidence regarding match running demands of both starters and non-starters. Nevertheless, most of the available studies on external load quantification in soccer need to consider starters and non-starters [2, 9]. It is known that, compared to non-starters, starter players usually present greater weekly loads for the overall distance-based measures. [10]. However, when considering a soccer match, a study conducted on 1066 professional soccer players showed that the non-starters covered greater high-intensity running distances than the starters [2].

Moreover, the analysis of the match running demands by 15 or 5 minutes has shown higher activity than the match average values [9]. Also, considering that the match running demands are position-dependent, it is paramount to examine the within- and between-players differences according to each player’s participation during matches as starters and non-starters. For instance, previous studies reported that increasing the time of a match duration passage decreased the intensity of the overall distance-based measures in all positions. In contrast, the differences among positions increased [11]. On the other hand, decreasing the time of a match duration passage to 5 minutes, no significant differences among positions are observed for high-speed running (HSR) and sprinting distances. This suggests that decreasing the time of the activity observation homogenizes the match running demands imposed on players [11].

To advance our understanding in this field, further research is required to investigate different distance-based measures covered per 5 minutes and to examine within-player and between-player differences based on playing position and type of match participation (starters vs. substitutes). It would be valuable to conduct a study that specifically focuses on understanding the intensities and locomotor demands of starters compared to substitutes, with the aim of determining whether fresh status could potentially enhance locomotor responses. Such research could provide valuable insights for coaches, assisting them in making informed decisions regarding the physical readiness and performance of players. By examining the impact of fresh status on locomotor responses, coaches may gain a better understanding of how to optimize player substitutions and manage player workload effectively.

For the above reasons, the present study aimed (i) to compare total distance, high-speed running (HSR) distance, and sprint distance covered per 5-minute epoch by players acting as both starters and substitutes; (ii) to compare the locomotor demands between the moments the players entered the match (45–60, 60–75 and 75–90 minutes); and (iii) to compare the locomotor demands of the players between the variations of the within- and between-playing positions.

## MATERIALS AND METHODS

### Experimental approach

We followed a longitudinal study design including sixteen professional male soccer team matches. During the study period, all theplayers were observed. However, for a repeated-measure design, to test starters vs. substitute players, regarding the locomotor demands, we only considered the players who acted as both starters and substitutes. The data collection was performed by a 5-minute epoch, representing the locomotor demands for each 5-minute split during the match. Considering that the substitute players entered only the second half of match play, the comparative analysis of locomotor demands between the starters and the substitutes focused only on this period of time of the matches.

### Participants

A single team was selected by convenience. A group of 21 outfield professional football male players (231 observations) from the first team of one of the Polish Ekstraklasa clubs (age: 24.9 ± 3.2 years, body height: 179.6 ± 5.5 cm, body weight: 76.1 ± 5 kg) participated in the research. Sixteen official matches were observed during the autumn season 2022/2023. The study followed the ethical standards for the study involving humans, as described in the Declaration of Helsinki and was approved for Medical Ethic Commity in Gdańsk (Decision number 62/2022). To ensure confidentiality, all data were anonymized before the analysis. The participants were preliminary informed about the study design and signed informed consent after being familiarized with the protocol.

### Match load monitoring

Over all the matches, the players used a GNSS unit (10 Hz, Vector S7, Catapult Innovations, Melbourne, Australia; 81 mm × 43 mm × 16 mm). In order to reduce inter-unit variability, the players always used the same device. The GNSS units were placed between the player’s shoulder blades and were activated according to a manufacturer’s guidelines before kick-off. To avoid potential unit differences, the players wore the same GNSS unit for each match [12]. The data recorded by the units were downloaded after each match for further analysis using Catapult OpenField Cloud Analytics (OpenField 3.9.0 Catapult Sports, Melbourne, Australia). The following variables were selected for the analysis during this study: field time, defined as the time spent on the field (FT; min), total distance (TD; m), distance in high-speed running, defined as running speed between 19.81–25.2 km/h (HSR; m) [13], sprint, defined as velocity greater than 25.2 km/h (SPR; m) [14], High Speed Running Count (HSRC) and Sprint Count (SC). The velocity thresholds chosen are those defined by both tracking system providers. All data from the Tracab system were provided by ChyronHego as a match report. The data from both systems (i.e., Catapult and Tracab) were extracted by 5-min epochs, i.e. splitting the official match time into 5 minute periods.

### Statistical procedures

The descriptive statistics were presented as mean, standard deviation, and 95% confidence intervals since the sample revealed non-homogeneity (Levene’s p < 0.005), and non-parametric tests were executed. As the same players, acting as both starters and substitutes, were compared, the Wilcoxon signed-rank test was used for comparing the locomotor demands. To make a comparison between playing positions, the Kruskal-Wallis test was applied, followed by the Mann-Whitney U test to conduct the pairwise comparisons. The r value was used as effect size, as suggested by previous studies [15]. The statistical procedures were executed in the SPSS software (version 28.0.0.0, IBM, Chicago, USA) for a p < 0.05.

## RESULTS

**FIG. 1:**
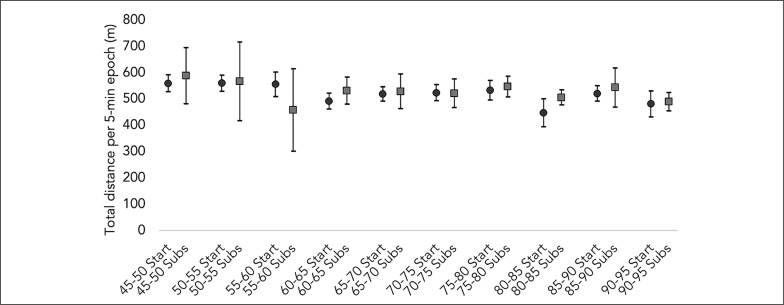
Descriptive statistics (average and 95% confidence interval) for total distance covered by starters and substitutes in 5-min epochs. Start: starters; Subs: substitutes.

**FIG. 2:**
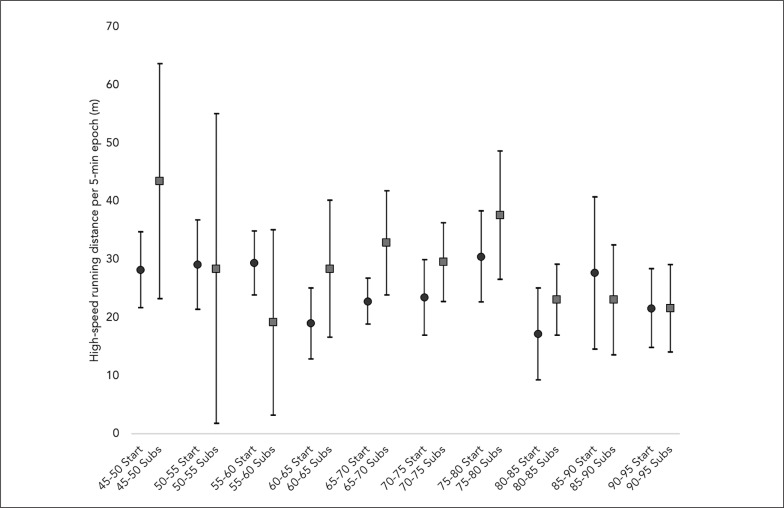
Descriptive statistics (average and 95% confidence interval) for high-speed running distance covered by starters and substitutes in 5-min epochs. Start: starters; Subs: substitutes.

**FIG. 3:**
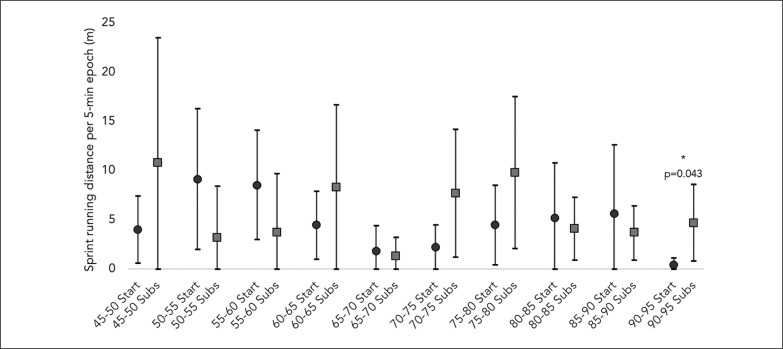
Descriptive statistics (average and 95% confidence interval) for sprint running distance covered by starters and substitutes in 5-min epochs. Start: starters; Subs: substitutes.

Figures 1, 2, and 3 describe the average and the 95% confidence intervals of total distance, HSR, and sprint distance covered in 5-min epochs for the players acting as both starters and substitutes. Over the second half of the matches, total distance covered by the starters during the average 5-min epochs was 523.2 ± 61.8 meters, while for substitutes it amounted to 522.9 ± 87.4 meters. Regarding HSR distance, the starters and the substitutes presented an average of 25.1 ± 27.8 and 27.8 ± 14.6 meters, respectively. Finally, the starters covered an average of 4.7 ± 7.2 meters for sprint distance, while the substitutes had an average of 5.8 ± 7.8 meters.

The Wilcoxon test comparisons between the starters and the substitutes regarding the demands performed in 5-min epochs over the second half of the match revealed no significant differences for total distance (Z = –1.225; p = 0.221), HSR distance (Z = –1.914; p = 0.056) and sprint distance (Z = –1.266; p = 0.206). Considering the comparisons between the splits over the second half, the only significant difference observed between the starters and the substitutes was for sprint distance in the 90–95 minute split (Z = –2.023; p = 0.043).

**FIG. 4:**
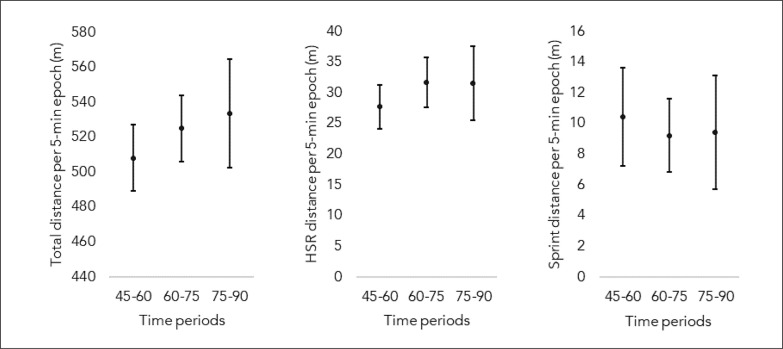
Descriptive statistics (average and 95% confidence interval) of the average locomotor demands considering the period (45–60 min; 60–75 min; 75–90 min) in which the substitute entered the match.

Figure 4 presents the descriptive statistics (average and 95% confidence interval) of the average locomotor demands considering the period (45–60 min; 60–75 min; 75–90 min) in which the substitute entered the match. No significant differences were found between the moment the substitute player entered the match for total distance (H = 2.650; p = 0.266), HSR distance (H = 1.738; p = 0.419) and sprint distance (H = 0.048; p = 0.976).

Table 1 presents the descriptive statistics (mean ± standard deviation) comparing the locomotor demands in 5-min epochs betweenand within-playing positions. The comparisons of between-playing positions revealed significant differences for total distance (H = 29.246; p < 0.001) and HSR distance (H = 12.153; p = 0.002) covered by the players acting as starters. Considering the substitutes, considerable differences between playing positions were found for HSR distance (H = 27.892; p < 0.001) and sprint distance (H = 15.879; p < 0.001). The starters playing in the position of midfielders covered significantly greater distances than external defenders (+15.9%; p < 0.001; r = –0.446) and forwards (+11.6%; p < 0.001; r = –0.278). Similarly, the distance covered by substitutes who played in the position ofmidfielders wassignificantly longer than the distance for external defenders (+26.1%; p < 0.001; r = –0.416) and forwards (+17.7%; p < 0.001; r = –0.499). Regarding the starters as forwards sprint distance covered was much longer than the distance covered by external defenders (+369.2%; p < 0.001; r = –0.446) and midfielders (+238.9%; p = 0.004; r = –0.211). Similarly, the substitutes playing in the position of forwards covered a significantly greater sprint distance than midfielders (+160.7%; p < 0.001; r = –0.402).

**TABLE 1:** Descriptive statistics (mean ± standard deviation) comparing locomotor demands in 5-min epochs between- and within-playing positions.

	External defenders	Midfielders	Forwards	Kruskal-Wallis test (Between playing positions)	Mann Whitney U p-value and r (effect size) between positions (pairwise comparison)
**Total distance at 5-min epoch (m)**					

Starters	485.8 ± 24.1	563.1 ± 82.5	504.4 ± 66.6	H = 29.246; p < 0.001	ED vs. MF: p < 0.001; r = –0.446
					ED vs. FW: p = 0.011; r = –0.215
					MF vs. FW: p < 0.001; r = –0.278

Substitutes	464.5 ± 124.6	585.9 ± 73.0	498.0 ± 76.8	H = 2.845; p = 0.241	ED vs. MF: p < 0.001; r = –0.416
					ED vs. FW: p = 0.253; r = –0.149
					MF vs. FW: p < 0.001; r = –0.499

Wilcoxon test for the within-playing position differences	Z = –0.454; p = 0.650	Z = –1.761; p = 0.078	Z = –0.221; p = 0.825		

**HSR distance at 5-min epoch (m)**					

Starters	18.8 ± 5.8	23.3 ± 10.9	24.7 ± 11.1	H = 12.153; p = 0.002	ED vs. MF: p = 0.176; r = –0.123
					ED vs. FW: p = 0.100; r = –0.139
					MF vs. FW: p = 0.678; r = –0.030

Substitutes	24.2 ± 12.8	31.2 ± 15.9	25.5 ± 14.4	H = 27.892; p < 0.001	ED vs. MF: p = 0.218; r = –0.155
					ED vs. FW: p = 0.621; r = –0.064
					MF vs. FW: p = 0.212; r = –0.127

Wilcoxon test for the within-playing position differences	Z = –1.293; p = 0.196	Z = –2.567; p = 0.010	Z = –0.295; p = 0.768		

**Sprint distance at 5-min epoch (m)**					

Starters	1.3 ± 3.4	1.8 ± 3.9	6.1 ± 6.9	H = 2.384; p = 0.304	ED vs. MF: p = 0.694; r = –0.036
					ED vs. FW: p = 0.006; r = –0.232
					MF vs. FW: p = 0.004; r = –0.211

Substitutes	4.7 ± 7.8	2.8 ± 4.8	7.3 ± 8.9	H = 15.879; p < 0.001	ED vs. MF: p = 0.253; r = –0.144
					ED vs. FW: p = 0.100; r = –0.214
					MF vs. FW: p < 0.001; r = –0.402

Wilcoxon test for the within-playing position differences	Z = –1.599; p = 0.110	Z = –1.372; p = 0.170	Z = –0.514; p = 0.607		

The within-playing positions analysis revealed no significant differences between the starters and the substitutes in any measures collected (p > 0.05).

## DISCUSSION

This article compared total distance, high-speed running (HSR) distance, and sprint distance covered per 5-minute epoch by the players acting both as starters and substitutes. Concerning this aim, some differences were reported only in the last time period (90–95 m), during which the substitutes out performed the starters in sprinting. The present study also aimed to compare the locomotor demands between the moments of entering the match (45–60, 60–75 and 75–90 minutes). The comparison showed that, the moment in which the substitution took place did not influence the dependent variables.

The current study also analized, the variations of the within- and between-playing positions regarding locomotor demands of the starters and the substitutes. The within-position differences were observed only for HSR among the midfielders, with higher values for the players acting as substitutes than for the starters. Finally, the between-position differences were also observed. The starter midfielders out performed external defenders and forwards in total distance. Similarly, the results were higher for the starters acting as forwards than as defenders. In addition, substitute forwards out performed substitute midfielders in sprinting.

Differences between starters and non-starters were extensively addressed in the soccer-related literature. For example, a previous study revelead that substitute players performed more high-intensity runs than starters [16]. Another study found higher workload demands of official matches for starters than for substitutes, although the non-starters out performed the starters when the variables were relativized by the actual playing time [17]. Interestingly, these acute match-related demands also impact on weekly workload, as was previously shown in the literature [18]. which reinforces the relevance of considering specific training programs for players from different groups (starters and substitutes). In the current study, the differences between starters and non-starters emerged only in the last period of the match. Specifically, the previous study did not examine when substitutes out performed starters, mostly indicating whether this had occurred. When a substitution takes place early in the second half of match play, fatigue-related detrimental effects are not supposed to commit starters, which can keep their regular performance fully. However, the group differences become more evident when the game reaches its final stages. This is similar to a previous study that reported higher sprinting and high-intensity running actions of substitutes compared to starters during the last 15 minutes of the game [19]. These differences were evident onlyin sprinting actions which are considered key to achieve success in soccer [20–22]. Therefore, having substitute players available at final stages of a match can prove to be advantageous for the team and can increase the probability of winning, especially considering an expected drop in physical performance during the match [23]. In summary, the current study indicates the relevance of substitutes and their role in keeping (or even improving) team’s performance during matches [24].

There were no differences in the physical demands between the substitutes that entered the game at different moments (considering 15-minute intervals). It could be expected that relativizing the variables for effective playing time would indicate a growing trend (i.e. higher responses for players entering the game at last stages) as players would be less exposed to fatigue effects, which was not observed. Two reasons can explain the current findings. First of all, previous studies showed no differences in physical responses of starters considering the 15-minute intervals during the first half of match play [19, 25]. Similar results were also observed in technical actions (e.g., passes, dribles), which do not seem to change over the first half for starters when considering different game periods [26]. Therefore, it seems that it takes longer than half-match (the time interval investigated in the current study) for between-player differences to emerge. Moreover, there is a growing number of substitutions over the second half of a match [27]. Along with other actions (injuries, winning teams stalling, and others), this recurrent game stop might reduce the physical advantage of a player recently called from the bench by giving the other players more time to recover. Therefore, time-related differences might be less evident, as shown in the current results. Finally, it is worth noting that previous research has indicated that substitutes, despite their relative physical impact, are unable to replicate or exceed the peak physical demands experienced by players who participate in the entire duration of a match [28]. This finding highlights the unique challenges and demands placed on players who are on the field for the full duration of a game.

Previous studies showed playing position-related differences in soccer, although not considering the role of a starter or a substitute was not considered. Interestingly, external defenders are among the most demanded players in official matches, presenting higher values of total distance and high-intensity actions than central midfielders and forwards [29], higher values of sprinting distance than central midfielders [30], and higher values of total distance and distance at the highest speed thresholds than central midfielders and forwards [31]. This trend, however, was not observed in the current research, as external defenders did not outperform other playing positions and, in fact, underperformed in total distance and sprinting. The main reason for this might be related to the characteristics of substitutions in elite soccer. Specifically, most substitutions have offensive purposes [16], and there is preference for replacing forwards. Therefore, external defenders might not be expected to be replaced in most matches, which might require them to save energy for the whole match, even unconsciously. On the other hand, forwards can adopt an all-out strategy when they believe they will be replaced in the second half. Consequently, the workload might be higher for them when considering the whole match, which explains the current results. To support this rationale, future studies should test whether providing players with explicit information about the substitutions before they occur during simulated matches will impact players’ match-related physical outcomes.

This study provides the first comprehensive assessment of the differences between starters and substitute players considering the time period and playing positions. Despite its strengths, such as the novelty, the size of the dataset, and the exciting results, there are some limitations that must be considered. First, teams are still adapting to the change of the rules relating to the number of substitutions allowed per match (from three to five). This implies that the current results might represent a transition between one and the other state, calling for follow-up designs to be confirmed. Moreover, the characteristics of the game model might influence the profile of the substitutions, which requires more contexts (e.g., leagues and clubs) to be investigated. The contextual factors (such as a match score, red cards, injuries, a match venue, and others) that might influence coaches’ decisions to replace a player were not considered and remained a gap in the literature. Finally, it is important to note that the effective playing time was not taken into consideration in this study, which may have an impact on the final outcomes [32]. Future research should address this limitation by focusing on analyzing the effective locomotor demands during the actual playing time, excluding pauses and breaks.

Future research is necessary to address the aforementioned limitations and further expand our understanding in this research area. To enhance the existing knowledge base, it is recommended that future studies compare different epoch periods, taking into consideration variations in playing positions and the effects of entering the field at different periods of the match. Additionally, incorporating more detailed tactical information rather than solely contextual information can provide a more comprehensive analysis and help elucidate the factors influencing variations in locomotor performance.

Despite the limitations of our study, it is worth noting (with caution and acknowledging the low certainty of evidence) that being substitute or starting the match does not appear to have a significant impact on locomotor performance. However, it is important to recognize that confounding variables, such as situational variables, tactical behavior, and team dynamics, can heavily influence locomotor responses. On the other hand, there is greater certainty in affirming that playing position significantly influences locomotor responses, and therefore, training interventions should consider this factor to adequately prepare players for the specific demands of their positions, irrespective of whether they are starters or substitutes.

## CONCLUSIONS

The substitutes out performed starters in sprinting, though the differences were observed only in the last time priod (90–95 m) The current study also compared the locomotor demands between the moments of entering the match (45–60, 60–75 and 75–90 minutes). The comparison showed that, the moment in which the substitution took place did not influence the dependent variables. Within-position differences were observed only for HSR among midfielders, with higher values for the substitutes than for the starters. Finally, betweenposition differences were observed in the current study. The starter midfielders out performed external defenders and forwards in total distance, which was also higher for the starter forwards compared to starter defenders. Moreover, the substitute forwards out performed substitute midfielders in sprinting.

## Ethics approval and consent to participate

Medical Ethic Commity in Gdańsk (Decision number 62/2022). All participants and their legal guardians were informed about the study and signed free consent.

## Consent for publication

Not applicable.

## Availability of Data and Materials

All data generated or analyzed during this study are available at the request of the corresponding author.

## Competing interests

The authors declare that they have no competing interests.
